# Assisted Living Care for Special Populations

**DOI:** 10.1093/geroni/igaa057.2350

**Published:** 2020-12-16

**Authors:** Philip Sloane, Sheryl Zimmerman

## Abstract

Assisted living (AL) is a notable provider of residential long-term care for older adults; there are almost twice as many AL communities as nursing homes, and they provide care to more than 800,000 older adults. As AL has evolved, it has come to serve more individuals with cognitive, mental, and health care needs. For example, 70% of residents have sleep disturbances, 42% have moderate/severe dementia, and mortality rates average 14% annually. Care needs include those for behaviors such as agitation, serious mental illness, and at the end-of-life. However, not all AL communities provide similar care. This symposium will use national data and data from a seven state study of 250 AL communities to focus on four populations receiving care in AL: persons with dementia, serious mental illness, sleep disturbances, and on hospice. The first speaker will discuss how AL staff conceive of and respond to behavioral expressions of persons with dementia; the second will focus on the use of psychosocial/environmental practices for persons with dementia in AL. The third speaker will discuss the growing proportion of persons with serious mental illness in AL and related implications for care. The fourth presenter will address the high use of melatonin in AL, as well as resident- and community-level correlates of melatonin prescribing. The final speaker will examine hospice use in AL and how it varies based on community characteristics. These findings related to care and care needs for four key populations have important implications for practice, policy, and future research.

